# The Endosymbiotic Coral Algae Symbiodiniaceae Are Sensitive to a Sensory Pollutant: Artificial Light at Night, ALAN

**DOI:** 10.3389/fphys.2021.695083

**Published:** 2021-06-21

**Authors:** Inbal Ayalon, Jennifer I. C. Benichou, Dror Avisar, Oren Levy

**Affiliations:** ^1^Mina and Everard Goodman Faculty of Life Sciences, Bar-Ilan University, Ramat Gan, Israel; ^2^Israel The H. Steinitz Marine Biology Laboratory, The Interuniversity Institute for Marine Sciences of Eilat, Eilat, Israel; ^3^Faculty of Exact Sciences, Porter School of the Environment and Earth Sciences, Tel Aviv University, Tel Aviv, Israel

**Keywords:** coral, light pollution, photosynthesis, Symbiodiniaceae, ALAN, light pollution

## Abstract

Artificial Light at Night, ALAN, is a major emerging issue in biodiversity conservation, which can negatively impact both terrestrial and marine environments. Therefore, it should be taken into serious consideration in strategic planning for urban development. While the lion’s share of research has dealt with terrestrial organisms, only a handful of studies have focused on the marine milieu. To determine if ALAN impacts the coral reef symbiotic algae, that are fundamental for sustainable coral reefs, we conducted a short experiment over a period of one-month by illuminating isolated Symbiodiniaceae cell cultures from the genera *Cladocopium* (formerly Clade C) and *Durusdinium* (formerly Clade D) with LED light. Cell cultures were exposed nightly to ALAN levels of 0.15 μmol quanta m^–2^ s^–1^ (∼4–5 lux) with three light spectra: blue, yellow and white. Our findings showed that even in very low levels of light at night, the photo-physiology of the algae’s Electron Transport Rate (ETR), Non-Photochemical Quenching, (NPQ), total chlorophyll, and meiotic index presented significantly lower values under ALAN, primarily, but not exclusively, in *Cladocopium* cell cultures. The findings also showed that diverse Symbiodiniaceae types have different photo-physiology and photosynthesis performances under ALAN. We believe that our results sound an alarm for the probable detrimental effects of an increasing sensory pollutant, ALAN, on the eco-physiology of symbiotic corals. The results of this study point to the potential effects of ALAN on other organisms in marine ecosystem such as fish, zooplankton, and phytoplankton in which their biorhythms is entrained by natural light and dark cycles.

## Introduction

The ever-increasing amount of nighttime light, known as Artificial Light at Night (ALAN), is an inherent consequence of population growth along ocean coastlines, and a major emerging sensory pollutant concern for coral reef ecosystems (Becker et al., 2013; Tamir et al., 2017; Ayalon et al., 2019, 2021). This is in addition to the other insults that these complex populations have been exposed to over the last few decades, either due to human actions (overfishing, chemical pollution), or natural stressors (storms, diseases, sedimentation, heatwaves), Baker et al. (2008), Dubinsky and Stambler (2010), Caroselli et al. (2017), Tamir et al. (2017), Hughes et al. (2018), Ayalon et al. (2019, 2021). The three-dimensional habitats that comprise a coral reef are among the most diverse marine ecosystems on earth, supporting thousands of organisms that live in close association and dependency. It is possible that sensory pollution could affect this ecosystem by altering neural processing, behavioral patterns, and endocrine signaling (Cucurachi, 2014; Halfwerk and Slabbekoorn, 2015). For example, ALAN could disrupt ecological behavior such as feeding time, active time (Bolton et al., 2017), reproduction, and communication by masking the natural light of the moon and stars and increasing the apparent periodicity of daily light hours (Bourgeois et al., 2009; Kaniewska et al., 2015; Swaddle et al., 2015). The natural day-night cycle is a crucial factor for many processes in the marine ecosystem, including metabolism, photosynthesis, night recovery, and hormone secretion, among other physiological and metabolic responses.

Broadcast spawning, the main mode of coral reproduction, depends on very precise timing of gamete release into the water column to be successful (Shlesinger and Loya, 1985). This is achieved by synchronization of the corals with the lunar cycle (Babcock et al., 1986; Kaniewska et al., 2015; Ayalon et al., 2021). Corals possess an array of sensitive photoreceptors, including GPCRs rhodopsins and cryptochromes, which operate in the blue region of the light spectrum (Gorbunov and Falkowski, 2002; Levy et al., 2007), and are synchronized with the moon phase. However, exogenous environmental factors, such as water temperature, low tides, or time after sunset, can also cue spawning timing (Harriott, 1983; Hunter, 1988; Wyers et al., 1991; Guest et al., 2002).

The relationship with the intracellular symbiont dinoflagellates (family Symbiodiniaceae) (LaJeunesse et al., 2018) is essential for corals to thrive in low-nutrient tropical oceans and, consequently, for the entire coral reef system. As their contribution to this symbiotic relationship, the endosymbionts provide most of the coral host’s metabolic energy requirements, enhancing calcification rates and coral growth (Muscatine and Porter, 1977). The role of symbiont diversity is key to coral survival under conditions that cause environmental bleaching or other stressors. Corals typically associate with Symbiodiniaceae from the genera *Symbiodinium* (formerly Clade A), *Breviolum* (formerly Clade B), *Cladocopium* (formerly Clade C), and *Durusdinium* (formerly Clade D), LaJeunesse et al. (2018), with each association being suitable for different environmental conditions such as irradiation, temperature, depth, and geographic location (Trench, 1993; Baker and Rowan, 1997; Rowan, 2004; Stat et al., 2008). The most common genera in symbiosis with corals are *Cladocopium* and *Symbiodinium*, while the association with *Durusdinium* is less common. Members of the latter genera display higher tolerance to thermal stress (Douglas, 1998; Hoegh-Guldberg, 1999).

The symbioses between corals and their endosymbiotic algae are strongly influenced by the surrounding environment (Douglas, 1998; Frade et al., 2008), and global or local multimodal pollutant stressors (Baker et al., 2008; Hughes et al., 2017) can impair the symbiotic relationship. The exponential rise in the anthropogenic stress of ALAN (Gaston et al., 2013; Davies et al., 2014; Tamir et al., 2017) resulting from population growth and the use of new electricity technology (Nicholls, 1995; Gaston et al., 2013; Davies et al., 2014) has prompted much research in this area in recent years (Davies et al., 2013; Tamir et al., 2017). We have previously described a strong impact of light at night on coral physiology, and the gametogenesis cycle and reproduction phylogeny (see Ayalon et al., 2019, 2021). Here we have extended these results by exploring the physiological and photosynthetic implications of light pollution by exposing two *Symbiodiniacea* cultures (*Cladocopium* and *Durusdinium*) to LED lights “cold white” (440–700 nm, 6,000–6,500 K) spectrum, and also to specific narrow light spectra (blue and yellow) that “mimic” the light spectrum and levels measured on shores close to coral reefs, in the Gulf of Eilat/Aqaba, which is an arm of the Red Sea (see Tamir et al., 2017). Our results clearly demonstrate that ALAN can influence *Symbiodiniacea* cultures, and that there are physiological differences in the responses of the two *Symbiodiniacea* alga types to light pollution.

## Materials and Methods

### Experimental Setup

*Symbiodiniacea* cell cultures of *Cladocopium* (CCMP 2466) and *Durusdinium* (CCMP 2556) in stationary growth stage were used in this study. All cell cultures were grown in 2 L Fernbach flasks containing 1 L half-strength medium (f/2) without silica. Illumination was provided by lateral white LED bulbs (440–700 nm, 6,000–6,500 K) under a 12L:12D cycle. Irradiance was measured by a quantum spherical sensor (LI-COR, Lincoln, NE, United States) and was 100 μmol quanta m^–2^ s^–1^ (4,400 lux). The cell cultures were maintained at 25°C in a culture room with controlled temperature during experiments. After 3 weeks of growth, the cell cultures were subdivided (100 ml/flask) and triplicates of flasks with each algae type were allocated to one of three experimental light groups, with an extra group acting as control. The cultures were treated with one of three colors of LED lights as previously published by our group (Ayalon et al., 2019, 2021): blue (420–480 nm, 10,000 K); yellow (580–620 nm, 2,000 K); or white (440–700 nm, 6,000–6,500 K), with an intensity of 0.15 μmol quanta m^–2^ s^–1^ (∼4–5 lux). An Ocean optics JAZ spectrometer was used to make the spectral measurements. Cell cultures were illuminated every night from 19:00 until 7:00 in the morning using a photocell, and during the daytime, all groups were illuminated with a white LED lamp as described above (440–700 nm, 6,000–6,500 K). The control group did not experience any light pollution at night, as a black curtain was used between treatments to avoid light contamination between the experimental groups. The cell cultures were sampled after one-month of exposure to light pollution for physiological and fluorescence assessments.

### Algae Density and Mitotic Index

The density of algae was measured on a 100-μl sample from each experimental treatment by counting cells on a Marienfeld hemacytometer. Cells were counted in five fields: all the corner squares plus the middle from each field, using a light microscope (Nikon Eclipse TE 2000-E; Nikon) under ×100 magnification. The average of the counts in each field was multiplied by 10,000 to give the numbers of cells/ml (each field is 0.1 cm × 0.1 cm × 0.01 cm depth). The same squares were counted to evaluate the Mitotic Index (MI), which is the ratio between the numbers of cells undergoing mitosis and the total number of cells.

### Algae Chlorophyll Measurements

In order to extract the chlorophyll from the symbiotic cells, 1 ml of each sample was centrifuged for 5 min at 2,000 *g* at 4°C. The pellet was then rinsed with 90% acetone and stored for 24 h at 4°C in the dark. The optical density (OD) at 630, 663, 750 nm was measured against a blank of 90% acetone using a Multiskan Spectrum Plate reader (Multiskan Spectrum; Thermo Scientific). The total concentration of chlorophyll (pg chlorophyll per cell) was calculated as described previously (Jeffrey and Humphrey, 1975).

### Fluorescence Measurements

Photosynthetic efficiency was measured in triplicates of experimental cell cultures using the IMAGING-PAM fluorescence (Pulse Amplitude modulation, Maxi-PAM, Walz Gmbh). The results were analyzed with the Imaging Win software program (v2.00; Walz Gmbh). In order to estimate photosynthetic performances, fluorescence yield, rapid light curve (RLC), electron transfer rate (ETR), and non-photochemical quenching (NPQ) were calculated after increasing the illumination at 20 sec intervals (0, 10, 35, 55, 110, 185, 230, 280, and 335; μmol quanta m^–2^ s^–1^). All samples were dark adapted for 40 min before measurements started.

### Statistical Analysis

Effects of light treatments on the number of symbionts, the mitotic index, and the total amount of chlorophyll were evaluated using a one-way ANOVA, followed by Tukey’s *post hoc* test. Homogeneity of variances and data normality were verified prior to the analysis by using Levene and Shapiro-Wilk tests, respectively. Results were expressed as mean ± standard error. Non–linear relationships between irradiance and ETR (or NPQ) under different light conditions were modeled with linear mixed-effects regressions. ETR (or NPQ) was defined as the outcome variable, and irradiance was defined as a continuous predictor. The light treatments were defined as a fixed factor, and the quantity of algae as a random effect. A third degree polynomial term for irradiance was included in the model, as well as an interaction between light treatments and irradiance, resulting in different curves for each light treatment. Several models were tested (with or without interactions, or with a lower polynomial degree) and the final models were selected based on the Akaike information criterion (AIC). Comparisons between light treatments were performed by pairwise comparisons of polynomial coefficients of the same degree. Statistical significance was assessed by testing linear contrasts and *p*-values were obtained by *Z*-tests and corrected for multiple comparisons using the FDR procedure (see Supplementary Tables 1–4 for detailed results). Homoscedasticity and the normality of residuals were inspected visually with residual plots. Regression analyses were conducted using the R statistical environment (R Core Team, 2020). R: A language and environment for statistical computing. R Foundation for Statistical Computing, Vienna, Austria URL^1^. Linear mixed models were fitted using the LmerTest package (Kuznetsova et al., 2017). *Post hoc* analysis with linear contrasts was performed using the *glht* function from the *multcomp* package (Hothorn et al., 2008).

## Results

**FIGURE 1:**
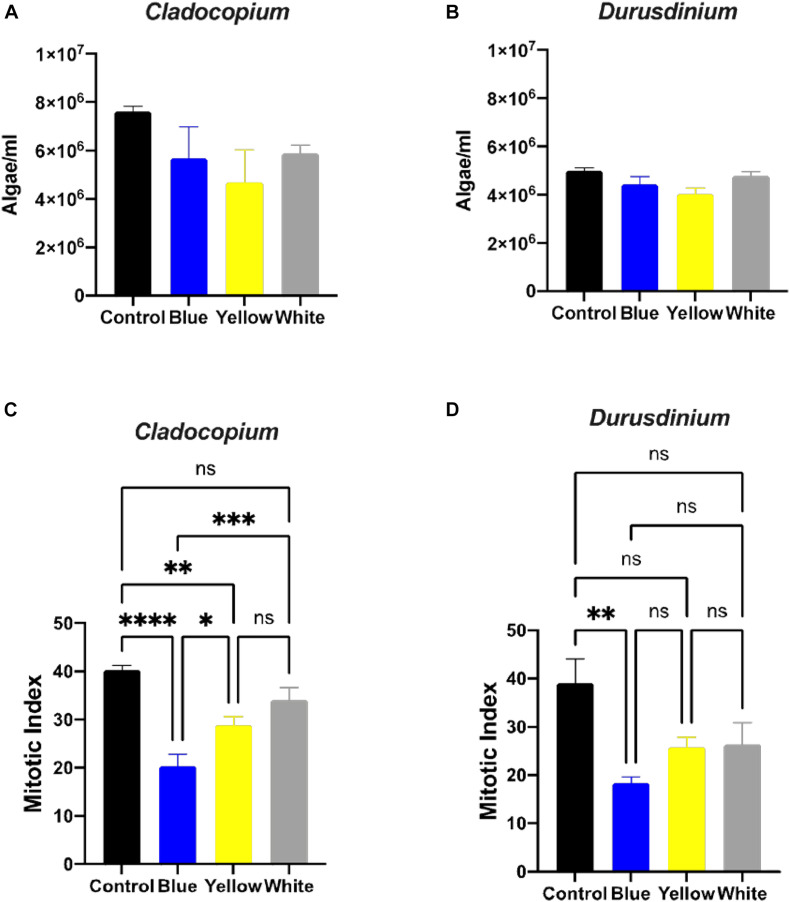
Algae growth and mitotic index ration of the two algae cultures exposed to ALAN. No significant differences in algae density in *Cladocopium* and *Durusdinium* cell cultures **(A,B)** after one-month exposure to ALAN resulting from blue, yellow or white LEDs; irradiance level 0.15 μmol quanta m^–2^ s^–1^ (4–5 lux). Data are expressed as mean ± SEM (*n* = 3). One way-Anova (*p* > 0.05) between control and different light treatments **(A,B)**. In contrast there are significant differences in the Mitotic Index (MI) **(C,D)**, Data are expressed as mean ± SEM (*n* = 3), ANOVA was followed by *post hoc* Tukey’s test, asterisks indicate significantly different mean values (*p < 0.05; **p < 0.01, ***p < 0.001, and ****p < 0.0001).

**FIGURE 2:**
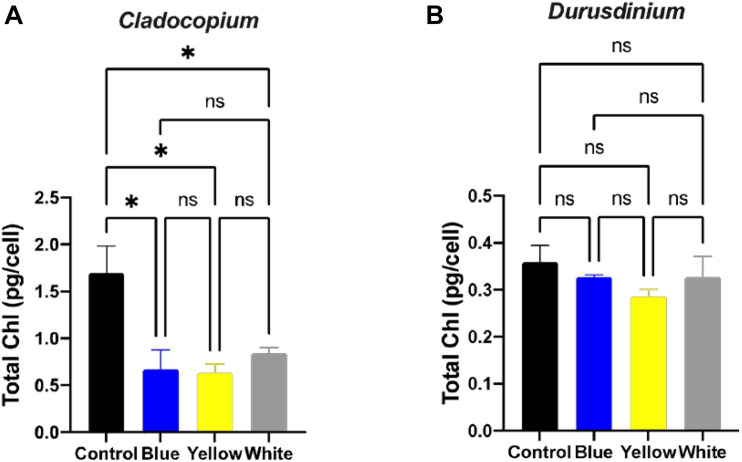
Total chlorophyll concentrations under ALAN. *Cladocopium* **(A)** and *Durusdinium* **(B)** cell cultures (pg total chl per ml) after one-month exposure to ALAN resulting from blue, yellow or white LEDs; irradiance level 0.15 μmol quanta m^–2^ s^–1^ (4–5 lux). Data are expressed as mean ± SEM (*n* = 3), ANOVA was followed by *post hoc* Tukey’s test, asterisks indicate significantly different mean values (*p < 0.05).

A number of parameters related to algae physiology were examined after one-month exposure to light pollution in order to evaluate the effect of ALAN on the symbiotic algae cell cultures. There was no significant effect of light conditions on the number of algae (*p* > 0.05) for either algal type, although the total number of cells per ml was higher for *Cladocopium* than *Durusdinium* cultures for all light treatments (Figures 1A,B). It should be noted that the starting concentrations were 1 million cells per ml for both types of algae. However, there was a significant reduction in the mitotic index of *Cladocopium* cultures after exposure to blue and yellow LED lights (*p* < 0.05) compared to the control, but no significant difference between control and exposure to white LED light (*p* > 0.05). In contrast the only significant difference in the mitotic index of *Durusdinium* cultures was between the control and the blue treatment (*p* < 0.001) with no difference observed with yellow or white light (*p* > 0.05, Figures 1C,D). Physiological analysis (Figure 2) revealed a significant reduction in total chlorophyll (pg total chl per ml) compared to the control in *Cladocopium* cultures after one-month exposure to ALAN (blue, yellow, and white LEDs, *p* < 0.05, Figure 2A). There were no such differences for *Durusdinium* cultures (Figure 2B).

**FIGURE 3:**
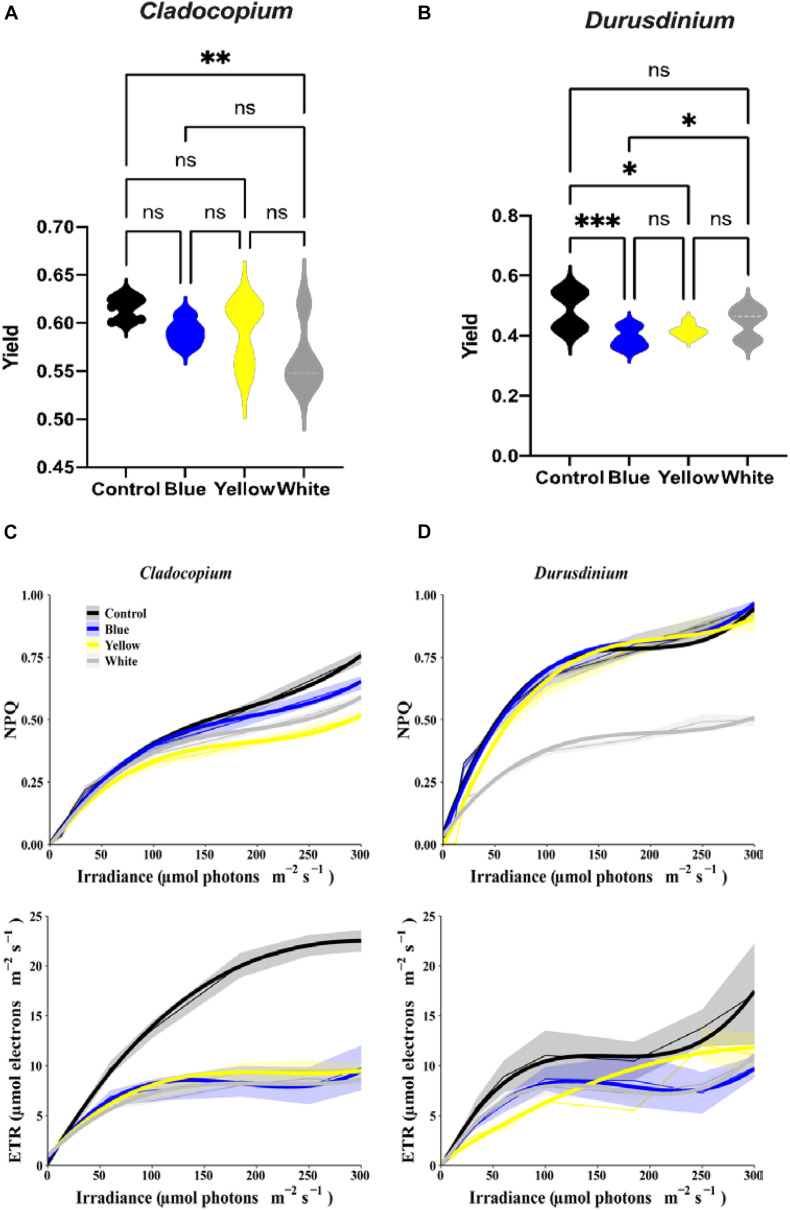
Relationship between irradiance fluorescence measurements after one-month exposure to ALAN resulting from blue, yellow or white LEDs; irradiance level 0.15 μmol quanta m^–2^ s^–1^ (4–5 lux). Fluorescence yield **(A,B)**, presented by violin plots with *n* = 12 measurements for each light treatment. ANOVA was followed by *post hoc* Tukey’s test, asterisks indicate significantly different mean values (*p < 0.05; **p < 0.01, and ***p < 0.001). Non-photochemical quenching NPQ (upper subplots) and ETR (lower subplots) for *Cladocopium* and *Durusdinium* **(C,D)**. Mean ETR (±SEM) for each irradiance level (left sub-plot). Thick lines represent the predicted fit obtained from 3rd degree polynomial linear mixed models. Thin lines represent mean NPQ (or ETR) ± SEM (represented by the ribbons).

The assay for maximal PSII Quantum Yield (*F*_*v*_/*F*_*m*_) performed after 40 min of darkness revealed significant differences between control and white treatments (*p* < 0.01) for the *Cladocopium* cells but no significant differences were induced by the other color lights (Figure 3A). In contrast, there were significant reductions in yield in the *Durusdinium* cell cultures after exposure to blue (*p* < 0.001), or yellow (*p* < 0.05) light, but not to white light (*p* > 0.05, see Figure 3B) compared to the relevant controls. As the next step, we modeled the non-linear relationship between ETR (and NPQ) and increasing irradiance levels under different light treatments with cubic polynomial linear mixed models (Figures 3C,D for *Cladocopium* and *Durusdinium*, respectively). The final models included interaction terms between irradiance and light treatment, resulting in a different regression model for each light treatment. For this reason, the models were compared by testing coefficients of same degree (see methods and supplementary material for results of pairwise comparisons of light treatments). The results revealed that all light treatments had a similar effect on NPQ in *Cladocopium* (the curves have a similar shape), although the distance between the curves slightly increases with rising irradiance levels, with the highest levels of NPQ in the control. The first-degree polynomial coefficients differed significantly between light treatments (*p* < 0.0001 for all comparisons), with the initial slope at an irradiance level of zero. In contrast, there were no differences between the control, blue, and yellow light for *Durusdinium* (most polynomial coefficients were not significantly different), while the white light treatment significantly reduced the NPQ compared to other light treatments (all polynomial coefficients were significantly different (*p* < 0.01), except for the third coefficient of the yellow light treatment). Regarding the effect of irradiance on ETR, the most significant effect of the light treatments was observed in *Cladocopium*, where the control ETR levels were significantly higher than all other light exposures (all polynomial coefficients, except the third degree, were significantly different, *p* < 0.005 for all comparisons).

All light treatments reduced ETR levels in *Durusdinium* but to a variable degree. Both blue and white light had the same effect on ETR (no significant differences between any coefficients). The control light treatment curve displays a similar shape to those of the blue and white lights, except an increased rate [first degree polynomial coefficient of the control was significantly different from those of the blue and white light (*p* < 0.0001)]. However, the yellow light treatment had a different effect; it generated a more gradual increase in ETR levels [3rd degree coefficients were significantly different between the yellow and other light treatments (*p* < 0.05 for all comparisons)].

## Discussion

In primary production chemical energy is converted into living biomass *via* photosynthesis, with light as the fundamental source. Since photosynthesis is usually closely linked to sunlight, the process is harmonically synchronized with daylight hours. While the photosynthesis machinery can theoretically function under irradiance levels slightly higher than moonlight (Raven and Cockell, 2006), and this may operate for algae adapted to long polar nights, the minimal thresholds for photosynthesis in nature have not been fully established (Grubisic, 2018). It is unclear whether light pollution can stimulate nocturnal photosynthesis, in this context, since the light typically supplied by street illumination is only 0.002% of daytime light levels (Grubisic, 2018). Light also serves as a universal cue in entraining the biological clock present in most living organisms on our planet to be harmonized with their environment. Therefore, altering the natural light regime by Artificial Light at Night, ALAN might impact the physiology of primary producers in complex and multiple ways, in addition to influencing photosynthesis.

Our results clearly show different responses of the algae cultures to light pollution which are genetically diverse. *Cladocopium* cell cultures were generally more sensitive to ALAN in comparison to *Durusdinium* cell cultures, This was observed as a significant decrease in the fluorescence yield under the white light exposer, a decrease in the mitotic index (MI) under blue and yellow light spectra, and a decrease in the total chlorophyll in all light treatments (Figures 1C, 2A, 3A). In the *Durusdinium* cell cultures there was a significant change in the MI decreasing only under the blue light spectra which was also more pronounced in the fluorescence yield values (Figures 1D, 3B). Both cell cultures showed no significant difference in the total number of algae per ml, although *Durusdinium* cell cultures grow less. The difference response of the two cell cultures is also aligned with previous studies on the eco-physiology of *Cladocopium* and *Durusdinium*. Little et al. (2004) showed that *Acropora* coral juveniles grow faster when associated with *Cladocopium* compared to *Acropora* juveniles associated with *Durusdinium*. The authors related the juveniles’ accelerated growth rate to a higher contribution of *Cladocopium* symbionts to the host nutrition related to faster rates of population growth inside the host (Little et al., 2004). Another observation resulted from our study was related to the performance of ETR of the two cell cultures. The control *Cladocopium* cell cultures showed higher ETR values when compared to *Durusdinium*, while both cell cultures had been affected with lower ETR values under ALAN. Nonetheless, this was far more evident and significant in *Cladocopium* cells (Figure 3). The decrease in photosynthetic capacities in our cell cultures is presumably due to oxidative stress as our previous research from our group examined corals exposed to ALAN showed a decrease in coral photosynthesis performances which was in line with oxidative stress (Ayalon et al., 2019; Levy et al., 2020) and even led to bleaching. The four coral species formerly tested under ALAN showed overproduction of reactive oxygen species (ROS) and an increase in oxidative damage to lipids in *Pocillopora damicornis*, *Acropora eurystoma*, and S*tylophora pistillata*, which experienced a more severe oxidative stress condition than the other species tested, *Turbinaria reniformis*. The question as to why *Durusdinium* cell cultures are less affected by ALAN may be related to lower phonesthetic capacity in the basal level as evident by the control ETR measurements, which probably lead to less ROS elevation and damage, or a more profound antioxidant scavenging system. This was shown in several types of *Symbiodiniacea* cultures. Among them, the most thermally sensitive was C*ladocopium*, which produced the highest amount of ROS at elevated temperatures, while *Durusdinium* cell cultures remain unaffected by elevated temperatures. This suggests that alternative mechanisms prevent ROS up-regulation in the first place, such as a more stable photosynthetic apparatus (McGinty et al., 2012).

Our study on *Symbiodiniacea* cultures is reported here for the first time. To the best of our knowledge, Poulin et al. (2014) was the first study to explore the effect of light pollution on diel changes in the photo-physiology of *Microcystis aeruginosa* (*freshwater cyanobacteria*). The researchers did this by mimicking the nearshore light conditions of a light−polluted area in the laboratory and investigating the effects on cyanobacteria cultures. They managed to show that nighttime irradiance levels comparable to nearshore light pollution in lakes can influence other aspects of the photo-physiology of *M. aeruginosa*. Thus, the study by Poulin et al. (2014) highlights the importance of looking specifically at the influence of artificial night lighting on phytoplankton. Their results revealed that while light with an irradiance level of six lux did not affect net photosynthesis or growth, it did influence photo−physiological parameters, such as photochemistry, functional absorption cross−section of photosystem II, and the RuBisCo number. Prolonged exposure to similar nocturnal illumination in the field was later reported by Hölker et al. (2015), who studied the influence of microbial communities in soft bottom sediments. In this case, artificial illumination changed the composition of the microbial community by increasing the numbers of primary producers, including diatoms and cyanobacteria (Hölker et al., 2015; Grubisic, 2018). Their results also indicated that ALAN could stimulate nocturnal photosynthesis at higher light intensities in the lab. Another study examined the microbiome associated with the reef-building coral *Acropora digitifera.* It investigated the temporal effects of ALAN on the coral-associated microbial community, and showed that some of the microbial taxa were significantly enriched in corals subjected to ALAN (Baquiran et al., 2020).

Considering, that ALAN is a new evolving area of study in marine ecosystems, our combined results on symbiotic algae cell cultures demonstrate the potential deleterious impacts of ALAN on phytoplankton and symbiotic corals exposed to increased light pollution, in particular blue and white light. As our worldwide-modeled light pollution atlas has shown, coral reefs in the Caribbean Sea, Red Sea, and places in the Pacific Ocean are under increasing light pollution (Ayalon et al., 2021). Therefore, we believe that ALAN can aggravate a decline in photosynthetic performance which will eventually led to deteriorated coral reefs. ALAN should now be considered as a chronic sensory pollutant which can lead to asynchronous in reproduction, decrease in growth rates and even bleaching. We hope that our results from this study can be used for urbane planning and mitigation of light pollution in coastal areas.

## Data Availability Statement

The original contributions presented in the study are included in the article/Supplementary Material, further inquiries can be directed to the corresponding author/s.

## Author Contributions

IA, OL, and DA: cell cultures growth, sampling processing, physiology analysis, photosynthesis measurements, manuscript writing, and data analyzing. JB, OL, and IA: statistical analysis. All authors contributed to the article and approved the submitted version.

## Conflict of Interest

The authors declare that the research was conducted in the absence of any commercial or financial relationships that could be construed as a potential conflict of interest.

## Footnotes

1 https://www.R-project.org/

## Supplementary Material

Supplementary Table 1 Summary of pairwise comparisons between different light treatments for each polynomial term obtained from cubic polynomial linear mixed modeling of non-linear relationship between NPQ and increasing irradiance levels, derived from Figure 3C for *Cladocopium.*

Supplementary Table 2 Summary of pairwise comparisons between different light treatments for each polynomial term obtained from cubic polynomial linear mixed modeling of non-linear relationship between ETR and increasing irradiance levels, derived from Figure 3C for *Cladocopium.*

Supplementary Table 3 Summary of pairwise comparisons between different light treatments for each polynomial term obtained from cubic polynomial linear mixed modeling of non-linear relationship between NPQ and increasing irradiance levels, derived from Figure 3D for *Durusdinium.*

Supplementary Table 4 Summary of pairwise comparisons between different light treatments for each polynomial term obtained from cubic polynomial linear mixed modeling of non-linear relationship between ETR and increasing irradiance levels, derived from Figure 3D for *Durusdinium*.
