# Supplementary material for: The Endosymbiotic Coral Algae Symbiodiniaceae Are Sensitive to a Sensory Pollutant: Artificial Light at Night, ALAN
Source: Front Physiol. 2021 Jun 21;12:695083. doi: 10.3389/fphys.2021.695083 (PMC8256845; doi:10.3389/fphys.2021.695083)
Supplement: Supplementary Table 1 — Summary of pairwise comparisons between different light treatments for each polynomial term obtained from cubic polynomial linear mixed modeling of non-linear relationship between NPQ and increasing irradiance levels, derived from Figure 3C for Cladocopium. [file Data_Sheet_1.docx]

Table S1: Summary of pairwise comparisons between different light treatments for each polynomial term obtained from cubic polynomial linear mixed modelling of nonlinear relationship between NPQ and increasing irradiance levels, derived from figure 3c for *Cladocopium.*

| **Polynomial degree** | **Comparison** | **Estimate**  **Difference** | **Standard Error** | \| **95% Confidence Interval of the Difference** \| \| --- \| | | **z value** | **P-value** |
| --- | --- | --- | --- | --- | --- | --- | --- | --- |
|  |  |  |  | **Lower** | **Upper** |  |  |
| 0 | Control - Blue | 0.0268 | 0.0194 | -0.0231 | 0.0767 | 1.3802 | 0.1675 |
|  | White - Blue | -0.028 | 0.0194 | -0.0779 | 0.0219 | -1.4413 | 0.1675 |
|  | Yellow - Blue | -0.0571 | 0.0194 | -0.107 | -0.0072 | -2.9417 | 0.0096 |
|  | White - Control | -0.0547 | 0.0194 | -0.1046 | -0.0048 | -2.8214 | 0.0096 |
|  | Yellow - Control | -0.0839 | 0.0194 | -0.1338 | -0.0339 | -4.3219 | 0.0001 |
|  | Yellow - White | -0.0291 | 0.0194 | -0.079 | 0.0208 | -1.5004 | 0.1675 |
| 1 | Control - Blue | 0.4719 | 0.0739 | 0.2823 | 0.6616 | 6.3866 | <0.0001 |
|  | White - Blue | -0.3444 | 0.0739 | -0.5341 | -0.1548 | -4.6607 | <0.0001 |
|  | Yellow - Blue | -0.7105 | 0.0739 | -0.9001 | -0.5209 | -9.6148 | <0.0001 |
|  | White - Control | -0.8164 | 0.0739 | -1.006 | -0.6267 | -11.0473 | <0.0001 |
|  | Yellow - Control | -1.1825 | 0.0739 | -1.3721 | -0.9928 | -16.0014 | <0.0001 |
|  | Yellow - White | -0.3661 | 0.0739 | -0.5557 | -0.1765 | -4.9541 | <0.0001 |
| 2 | Control - Blue | 0.1855 | 0.0739 | -0.0042 | 0.3753 | 2.5106 | 0.0723 |
|  | White - Blue | 0.0552 | 0.0739 | -0.1346 | 0.2449 | 0.7468 | 0.5131 |
|  | Yellow - Blue | 0.1035 | 0.0739 | -0.0863 | 0.2933 | 1.4008 | 0.3226 |
|  | White - Control | -0.1303 | 0.0739 | -0.3201 | 0.0594 | -1.7638 | 0.2333 |
|  | Yellow - Control | -0.082 | 0.0739 | -0.2718 | 0.1078 | -1.1099 | 0.4006 |
|  | Yellow - White | 0.0483 | 0.0739 | -0.1414 | 0.2381 | 0.654 | 0.5131 |
| 3 | Control - Blue | -0.0185 | 0.0739 | -0.2083 | 0.1713 | -0.2504 | 0.9178 |
|  | White - Blue | 0.0225 | 0.0739 | -0.1674 | 0.2123 | 0.3039 | 0.9178 |
|  | Yellow - Blue | -0.0109 | 0.0739 | -0.2007 | 0.1789 | -0.1472 | 0.9178 |
|  | White - Control | 0.041 | 0.0739 | -0.1489 | 0.2308 | 0.5543 | 0.9178 |
|  | Yellow - Control | 0.0076 | 0.0739 | -0.1822 | 0.1974 | 0.1033 | 0.9178 |
|  | Yellow - White | -0.0333 | 0.0739 | -0.2231 | 0.1565 | -0.451 | 0.9178 |

Table S2: Summary of pairwise comparisons between different light treatments for each polynomial term obtained from cubic polynomial linear mixed modelling of nonlinear relationship between ETR and increasing irradiance levels, derived from figure 3c for *Cladocopium.*

| **Polynomial degree** | **Comparison** | **Estimate**  **Difference** | **Standard Error** | \| **95% Confidence Interval of the Difference** \| \| --- \| | | | **z value** | **P-value** |
| --- | --- | --- | --- | --- | --- | --- | --- | --- | --- |
|  |  |  |  | **Lower** | **Upper** | |  |  |
| 0 | Control - Blue | 5.2544 | 0.6358 | 3.622 | | 6.8867 | 8.2639 | <0.0001 |
|  | White - Blue | -0.3424 | 0.6362 | -1.9756 | | 1.2909 | -0.5382 | 0.7085 |
|  | Yellow - Blue | 0.1948 | 0.6367 | -1.4397 | | 1.8293 | 0.306 | 0.7596 |
|  | White - Control | -5.5967 | 0.5683 | -7.0558 | | -4.1377 | -9.8479 | <0.0001 |
|  | Yellow - Control | -5.0595 | 0.5689 | -6.52 | | -3.599 | -8.894 | <0.0001 |
|  | Yellow - White | 0.5372 | 0.5693 | -0.9243 | | 1.9987 | 0.9437 | 0.518 |
| 1 | Control - Blue | 76.0805 | 3.5206 | 67.0462 | | 85.1148 | 21.6102 | <0.0001 |
|  | White - Blue | -0.528 | 3.5648 | -9.6757 | | 8.6197 | -0.1481 | 0.8822 |
|  | Yellow - Blue | 4.4368 | 3.6277 | -4.8723 | | 13.746 | 1.2231 | 0.2656 |
|  | White - Control | -76.6085 | 3.0998 | -84.563 | | -68.654 | -24.7141 | <0.0001 |
|  | Yellow - Control | -71.6437 | 3.1719 | -79.7833 | | -63.504 | -22.5868 | <0.0001 |
|  | Yellow - White | 4.9649 | 3.2209 | -3.3004 | | 13.2302 | 1.5415 | 0.1848 |
| 2 | Control - Blue | -13.0248 | 3.5209 | -22.0711 | | -3.9786 | -3.6993 | 0.0004 |
|  | White - Blue | 1.6876 | 3.5586 | -7.4556 | | 10.8308 | 0.4742 | 0.7354 |
|  | Yellow - Blue | -1.2207 | 3.6124 | -10.5022 | | 8.0609 | -0.3379 | 0.7354 |
|  | White - Control | 14.7125 | 3.1073 | 6.7288 | | 22.6961 | 4.7348 | <0.0001 |
|  | Yellow - Control | 11.8042 | 3.1688 | 3.6625 | | 19.9459 | 3.7251 | 0.0004 |
|  | Yellow - White | -2.9083 | 3.2107 | -11.1576 | | 5.341 | -0.9058 | 0.5476 |
| 3 | Control - Blue | -10.1687 | 3.5214 | -19.2092 | | -1.1282 | -2.8877 | 0.0233 |
|  | White - Blue | -6.3751 | 3.5455 | -15.4773 | | 2.7272 | -1.7981 | 0.1443 |
|  | Yellow - Blue | -6.4539 | 3.5799 | -15.6446 | | 2.7369 | -1.8028 | 0.1443 |
|  | White - Control | 3.7937 | 3.1231 | -4.2243 | | 11.8116 | 1.2147 | 0.2881 |
|  | Yellow - Control | 3.7148 | 3.1622 | -4.4034 | | 11.8331 | 1.1748 | 0.2881 |
|  | Yellow - White | -0.0788 | 3.1889 | -8.2658 | | 8.1081 | -0.0247 | 0.9803 |

Table S3: Summary of pairwise comparisons between different light treatments for each polynomial term obtained from cubic polynomial linear mixed modelling of nonlinear relationship between NPQ and increasing irradiance levels, derived from figure 3d for *Durusdinium.*

| **Polynomial degree** | **Comparison** | **Estimate**  **Difference** | **Standard Error** | \| **95% Confidence Interval of the Difference** \| \| --- \| | | **z value** | **P-value** |
| --- | --- | --- | --- | --- | --- | --- | --- | --- |
|  |  |  |  | **Lower** | **Upper** |  |  |
| 0 | Control - Blue | 0.0001 | 0.0218 | -0.0559 | 0.0561 | 0.0051 | 0.9959 |
|  | White - Blue | -0.2296 | 0.0218 | -0.2857 | -0.1736 | -10.5261 | <0.0001 |
|  | Yellow - Blue | -0.0449 | 0.0218 | -0.1009 | 0.0112 | -2.056 | 0.0477 |
|  | White - Control | -0.2297 | 0.0218 | -0.2858 | -0.1737 | -10.5312 | <0.0001 |
|  | Yellow - Control | -0.045 | 0.0218 | -0.101 | 0.0111 | -2.0611 | 0.0477 |
|  | Yellow - White | 0.1848 | 0.0218 | 0.1288 | 0.2408 | 8.4701 | <0.0001 |
| 1 | Control - Blue | -0.2642 | 0.1463 | -0.6403 | 0.112 | -1.8053 | 0.0852 |
|  | White - Blue | -2.1081 | 0.1463 | -2.4842 | -1.732 | -14.4069 | <0.0001 |
|  | Yellow - Blue | 0.207 | 0.1463 | -0.1692 | 0.5831 | 1.4145 | 0.1572 |
|  | White - Control | -1.8439 | 0.1463 | -2.2201 | -1.4678 | -12.6016 | <0.0001 |
|  | Yellow - Control | 0.4711 | 0.1463 | 0.095 | 0.8473 | 3.2198 | 0.0019 |
|  | Yellow - White | 2.3151 | 0.1463 | 1.9389 | 2.6912 | 15.8214 | <0.0001 |
| 2 | Control - Blue | 0.0494 | 0.1463 | -0.3263 | 0.4252 | 0.3377 | 0.7356 |
|  | White - Blue | 0.6915 | 0.1463 | 0.3158 | 1.0673 | 4.7259 | <0.0001 |
|  | Yellow - Blue | -0.2044 | 0.1463 | -0.5802 | 0.1713 | -1.3971 | 0.1949 |
|  | White - Control | 0.6421 | 0.1463 | 0.2664 | 1.0178 | 4.3882 | <0.0001 |
|  | Yellow - Control | -0.2539 | 0.1463 | -0.6296 | 0.1219 | -1.7348 | 0.1242 |
|  | Yellow - White | -0.896 | 0.1463 | -1.2717 | -0.5202 | -6.123 | <0.0001 |
| 3 | Control - Blue | 0.0939 | 0.1463 | -0.2822 | 0.4701 | 0.642 | 0.5209 |
|  | White - Blue | -0.4717 | 0.1463 | -0.8478 | -0.0956 | -3.2236 | 0.0038 |
|  | Yellow - Blue | -0.197 | 0.1463 | -0.5731 | 0.1792 | -1.3461 | 0.2139 |
|  | White - Control | -0.5656 | 0.1463 | -0.9418 | -0.1895 | -3.8656 | 0.0007 |
|  | Yellow - Control | -0.2909 | 0.1463 | -0.667 | 0.0852 | -1.9881 | 0.0907 |
|  | Yellow - White | 0.2747 | 0.1463 | -0.1014 | 0.6509 | 1.8775 | 0.0907 |

Table S4: Summary of pairwise comparisons between different light treatments for each polynomial term obtained from cubic polynomial linear mixed modelling of nonlinear relationship between ETR and increasing irradiance levels, derived from figure 3d for *Durusdinium.*

| **Polynomial degree** | **Comparison** | **Estimate**  **Difference** | **Standard Error** | \| **95% Confidence Interval of the Difference** \| \| --- \| | | **z value** | **P-value** |
| --- | --- | --- | --- | --- | --- | --- | --- | --- |
|  |  |  |  | **Lower** | **Upper** |  |  |
| 0 | Control - Blue | 1.9166 | 0.769 | -0.0618 | 3.895 | 2.4923 | 0.0347 |
|  | White - Blue | 0.0943 | 0.7662 | -1.8769 | 2.0654 | 0.123 | 0.9021 |
|  | Yellow - Blue | -0.2275 | 0.7688 | -2.2054 | 1.7505 | -0.2959 | 0.9021 |
|  | White - Control | -1.8223 | 0.766 | -3.793 | 0.1483 | -2.379 | 0.0347 |
|  | Yellow - Control | -2.1441 | 0.7687 | -4.1216 | -0.1666 | -2.7893 | 0.0317 |
|  | Yellow - White | -0.3217 | 0.7658 | -2.292 | 1.6485 | -0.4201 | 0.9021 |
| 1 | Control - Blue | 25.1911 | 4.8178 | 12.8107 | 37.5715 | 5.2287 | <0.0001 |
|  | White - Blue | 1.4434 | 4.9523 | -11.2825 | 14.1693 | 0.2915 | 0.7707 |
|  | Yellow - Blue | 15.6615 | 5.0105 | 2.7861 | 28.5369 | 3.1258 | 0.0035 |
|  | White - Control | -23.7477 | 4.5266 | -35.3797 | -12.1158 | -5.2463 | <0.0001 |
|  | Yellow - Control | -9.5296 | 4.5902 | -21.325 | 2.2657 | -2.0761 | 0.0455 |
|  | Yellow - White | 14.2181 | 4.7311 | 2.0606 | 26.3756 | 3.0053 | 0.004 |
| 2 | Control - Blue | 2.7374 | 4.7655 | -9.5058 | 14.9807 | 0.5744 | 0.6788 |
|  | White - Blue | 4.1913 | 4.6323 | -7.7099 | 16.0926 | 0.9048 | 0.5484 |
|  | Yellow - Blue | 11.171 | 4.98 | -1.6234 | 23.9654 | 2.2432 | 0.1493 |
|  | White - Control | 1.4539 | 4.5969 | -10.3562 | 13.264 | 0.3163 | 0.7518 |
|  | Yellow - Control | 8.4335 | 4.947 | -4.2761 | 21.1432 | 1.7048 | 0.2647 |
|  | Yellow - White | 6.9796 | 4.8189 | -5.4009 | 19.3602 | 1.4484 | 0.295 |
| 3 | Control - Blue | 5.7278 | 4.7461 | -6.4547 | 17.9103 | 1.2068 | 0.3412 |
|  | White - Blue | 2.4557 | 4.7748 | -9.8004 | 14.7119 | 0.5143 | 0.607 |
|  | Yellow - Blue | -14.7773 | 5.1724 | -28.0539 | -1.5006 | -2.8569 | 0.0086 |
|  | White - Control | -3.2721 | 4.5071 | -14.841 | 8.2969 | -0.726 | 0.5614 |
|  | Yellow - Control | -20.5051 | 4.9263 | -33.15 | -7.8601 | -4.1624 | 0.0002 |
|  | Yellow - White | -17.233 | 4.954 | -29.949 | -4.517 | -3.4786 | 0.0015 |
